# Genetic Monogamy and Low Reproductive Skew in a Disease‐Impacted, Communal‐Breeding Frog

**DOI:** 10.1002/ece3.74510

**Published:** 2026-10-22

**Authors:** Alexander S. Wendt, Anthony W. Waddle, Marcus A. Hough, Lee Berger, Lee F. Skerratt, Laura A. Brannelly

**Affiliations:** ^1^ Melbourne Veterinary School, Faculty of Science The University of Melbourne Werribee Victoria Australia; ^2^ Applied Biosciences Macquarie University Sydney New South Wales Australia

**Keywords:** amphibian breeding ecology, *Batrachochytrium dendrobatidis*, effective population size, genetic monogamy, mating system, parentage analysis, reproductive skew

## Abstract

Genetic mating systems shape reproductive skew, mate choice, and effective population size, yet remain poorly understood in many communally breeding amphibians. We used genetic parentage analysis to examine mating structure in the alpine tree frog (*Litoria verreauxii alpina*), a *Batrachochytrium dendrobatidis* (*Bd*) impacted amphibian that breeds in communal aggregations. Over a 6‐week breeding season, we recorded 477 capture events involving 197 unique adults and observed 55 egg masses. Using DArTseq‐derived SNP markers and COLONY, we reconstructed mating pairs, assessed male breeding success, and estimated effective population size (*N*_*e*_). Of the 45 egg masses with at least one successfully genotyped embryo, 97.7% had a definitive sire assigned, and every egg mass with more than one genotyped embryo was assigned to a single sire. Only one sampled male and one inferred female showed evidence of multiple mates, indicating a predominantly genetically monogamous mating structure despite communal breeding. Male reproductive success was not predicted by body size, recapture frequency, or breeding‐season timing. Effective population size was low, suggesting ongoing genetic vulnerability, but relatively even reproductive contribution among breeders may help limit reproductive skew. These findings contrast with expectations for communal‐breeding frogs, where high breeding‐site encounter rates and male‐biased attendance can create opportunities for multiple mating and unequal reproductive success. More broadly, these results show how genetic parentage data can reveal unexpected mating‐system structure in disease‐impacted amphibian populations.

## Graphical abstract

Genetic mating systems can shape reproductive skew and effective population size, yet remain poorly understood in many communally breeding amphibians. Using SNP‐based parentage analysis in the alpine tree frog ( *Litoria verreauxii* *alpina*), we found a predominantly genetically monogamous mating structure, with 97.7% of genotyped egg masses assigned to a sire and all clutches assigned to a single sire. Together, these results reveal unexpectedly limited multiple mating in a communal‐breeding amphibian and provide insight into how reproductive contribution is distributed under disease‐associated demographic pressure.

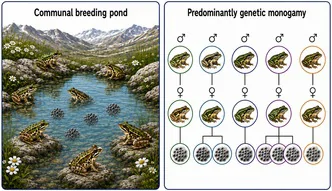

## 1 Introduction

Animals exhibit a remarkable diversity of mating systems, each distinctly shaping the genetic landscape of populations. By determining which individuals reproduce, how often they reproduce, and whether offspring are produced with one or multiple partners, mating systems influence reproductive skew, sexual selection, and realized parentage (Avise et al. 2002; Shuster 2009; Godwin et al. 2020). Reproductive strategies influence genetic diversity, adaptability, and evolutionary potential. Monogamy can promote stability and parental investment but may limit genetic variation and adaptive capacity if partners are closely related (Schaedelin et al. 2015; Winternitz et al. 2015). Polygyny, where one male mates with multiple females, often reduces genetic diversity due to reproductive skew among dominant males (Ptak and Lachmann 2003; Reuther 2009; Fitzpatrick and Servedio 2017; Angom et al. 2020; Singh et al. 2022; Elsner‐Gearing et al. 2024). In contrast, polyandry, which refers to a single female mating with multiple males, can increase genetic variability among offspring. However, it imposes energetic costs on females and can intensify male–male competition both through direct contests for mates and post‐copulatory sperm competition (Holman and Kokko 2013; Reding 2015; Zamudio et al. 2016; Godwin et al. 2020). Polygynandry, in which both sexes have multiple mates, may reduce inbreeding and enhance resilience to environmental pressures, but it can also intensify reproductive competition and increase mate‐searching effort (Baker et al. 2004; Cooper et al. 2011; Wang et al. 2020). Thus, mating systems provide an important framework for understanding how behavioral interactions translate into patterns of parentage, reproductive success, and genetic structure in natural populations (Avise et al. 2002; O'Brien et al. 2018; Wang et al. 2020). These links are especially important in small or demographically constrained populations, where modest differences in reproductive success can strongly influence effective population size and the distribution of genetic contribution among breeders (Hedrick 2005; Hare et al. 2011; Waples 2022).

Although a species can appear to have characteristics of a particular mating system, genetic analysis can reveal a more complex or entirely different structure (Cohas and Allainé 2009; O'Brien et al. 2018; Hoogland et al. 2019). Distinguishing between social and genetic mating systems is crucial for understanding the reproductive strategies, gene flow, and evolutionary dynamics occurring within a species. This is particularly relevant in amphibians, which exhibit one of the most diverse arrays of mating systems among vertebrates (Kühnel et al. 2010; Vági et al. 2020; Vági and Székely 2023). Amphibian reproductive strategies range from explosive breeding aggregations and prolonged mate guarding to post‐mating clutch piracy, sperm storage and even forms of parental care including egg attendance and tadpole transport (Sever 2002; Tennessen and Zamudio 2003; Vieites et al. 2004; Brown et al. 2010; Kuehnel and Kupfer 2012; de Sá et al. 2020; Rueda‐Solano et al. 2022; Gould and Valdez 2023). Together, these patterns highlight the need to investigate both observable social behaviors and the underlying genetic outcomes to fully understand the forces shaping amphibian mating systems. Such an integrated approach helps reveal the sexual selection traits and reproductive strategies that influence mate choice, genetic diversity, and population dynamics (Avise et al. 2002; O'Brien et al. 2018). These same processes are also central to effective population size (*N*_*e*_), which reflects the number of individuals that successfully contribute genes to the next generation rather than simply the total number of adults present. Because *N*_*e*_ is shaped by variance in reproductive success, skewed sex ratios, and unequal mating opportunities, it provides an important link between breeding structure and the retention or loss of genetic diversity (Avise et al. 2002; Briggs et al. 2005; Hare et al. 2011; O'Brien et al. 2018; Waples 2022). For species experiencing demographic decline, disease, or environmental stress, genetic mating systems may therefore provide insight into whether reproductive opportunities are concentrated among a few individuals or distributed more evenly across breeders.

The alpine tree frog (*Litoria verreauxii alpina*) provides a useful system for examining these relationships. The species breeds in dense communal aggregations in shallow alpine ponds over a short breeding season, creating frequent opportunities for interactions among potential mates (Brannelly et al. 2015, 2024; Anstis 2017). Male attendance at breeding sites is strongly biased relative to female attendance, with males often remaining at breeding ponds for extended periods while females generally attend for shorter periods (Brannelly et al. 2015). Such high encounter rates and male‐biased breeding‐site attendance create substantial opportunities for multiple mating and might therefore be expected to produce a polygamous mating structure and unequal reproductive success (Wells 2007; Zamudio et al. 2016). However, the genetic mating system of *L. v. alpina* has not previously been characterized and it is unknown whether the communal breeding behavior results in multiple mating and high reproductive skew or whether reproductive success is distributed more evenly across breeders.

The contemporary breeding structure of *L. v. alpina* has been substantially reshaped by endemic *Batrachochytrium dendrobatidis* (*Bd*) among the remnant populations. Disease‐driven changes in population size, age structure, and sex ratios can influence mate availability, reproductive opportunities, and reproductive skew (Addis et al. 2015; Beranek et al. 2022; Lampo et al. 2023). Within *L. v. alpina*, *Bd* causes high adult mortality, shortened adult lifespans, and greatly reduced generational overlap, and because females mature later than males, these demographic changes might alter mate availability and reproductive opportunities (Brannelly et al. 2015; Scheele et al. 2015, 2016; Beranek et al. 2022). Although we do not examine individual infection status in this study, these disease‐associated demographic changes provide important context for understanding the species' contemporary mating structure.

Understanding how reproductive opportunities are distributed among individuals and across the breeding season is important for interpreting population persistence. In amphibians, observed breeding behavior does not always reflect realized genetic mating patterns, and reproductive success can vary substantially among individuals and through time (Avise et al. 2002; O'Brien et al. 2018; de Sá et al. 2020). Seasonal changes in reproductive allocation may also be important where breeding occurs over a short period and environmental conditions change rapidly; yet these within‐season patterns remain poorly understood for many amphibians (Lips 2001; Jourdan‐Pineau et al. 2022).

Here, we characterize the genetic mating system of *L. v. alpina* and examine how reproductive output and mating success vary across its breeding season. We also estimate effective population size to place these reproductive patterns in a broader genetic context. Together, this study addresses important gaps in the breeding ecology of *L. v. alpina* and provides insight into how mating structure and seasonal reproductive allocation are expressed in a disease‐impacted amphibian population.

## 2 Methods

### 2.1 Study Population

This study was conducted on a population of alpine tree frogs (*Litoria verreauxii alpina*) located in the Victorian Alps of southeastern Australia. The species occurs in alpine and sub‐alpine habitats of southeastern New South Wales and Victoria, generally above 1200 m elevation (Office of Environment and Heritage (NSW) 2012). *Litoria v. alpina* is an aggregate breeder that reproduces in both natural and artificial wetlands including ponds, bogs, streamside pools, and drainage channels containing still or slow‐flowing water (Gillespie et al. 1995; Anstis 2017). Breeding occurs during the early spring snowmelt and typically lasts approximately 6–12 weeks (Brannelly et al. 2015, 2020; Brannelly, Hunter, et al. 2016). Eggs develop rapidly and typically hatch within 1 week of oviposition (Hunter et al. 1998; Osborne et al. 1999).

The focal breeding pond sampled is a permanent, manmade waterbody with an area of 7.1 m^2^, located approximately 1600 m above sea level. Although permanent, the pond does experience water loss through the breeding season and is also subject to disturbance associated with road maintenance for recreational off‐roading. Sampling occurred weekly for 6 weeks during the 2018 breeding season. The data analyzed here were part of a larger study exploring the impacts of infection on the Victorian populations of *L. v. alpina* (Brannelly et al. 2025).

### 2.2 Data Collection

We captured frogs by hand using a fresh pair of gloves for each individual and immediately placed the frogs in clean zipper‐seal plastic bags. Upon initial capture, we recorded the sex and collected morphometric data, including snout‐vent length (measured to the nearest 0.01 mm) and mass (measured to the nearest 0.1 g). We photographed each frog on the dorsal surface and right side of the groin using a Panasonic DMC‐G5 camera with a Lumix G VARIO 14–42 mm F3.5–5.6 lens. Individuals were identified on subsequent captures by visually comparing dorsal and groin markings with photographs collected during previous captures (Figure 1). Photographic identification based on natural body markings is the standard for this species (Brannelly et al. 2015; Brannelly, Hunter, et al. 2016) because other methods of tagging have a high failure rate (Brannelly et al. 2014). This method has also been evaluated in other Australian *Litoria* species (Kenyon et al. 2009; Crawford‐Ash and Rowley 2021). Upon first capture, we collected toe tip clips for DNA extraction and subsequent parentage analysis. Toe tip clips were obtained using bleach‐sterilized scissors and stored in tubes containing 100% ethanol. Each time an animal was recaptured we repeated the collection of morphometric data and compared it with initial photographs but only took a toe‐tip sample upon first capture (one for each animal captured).

**FIGURE 1:**
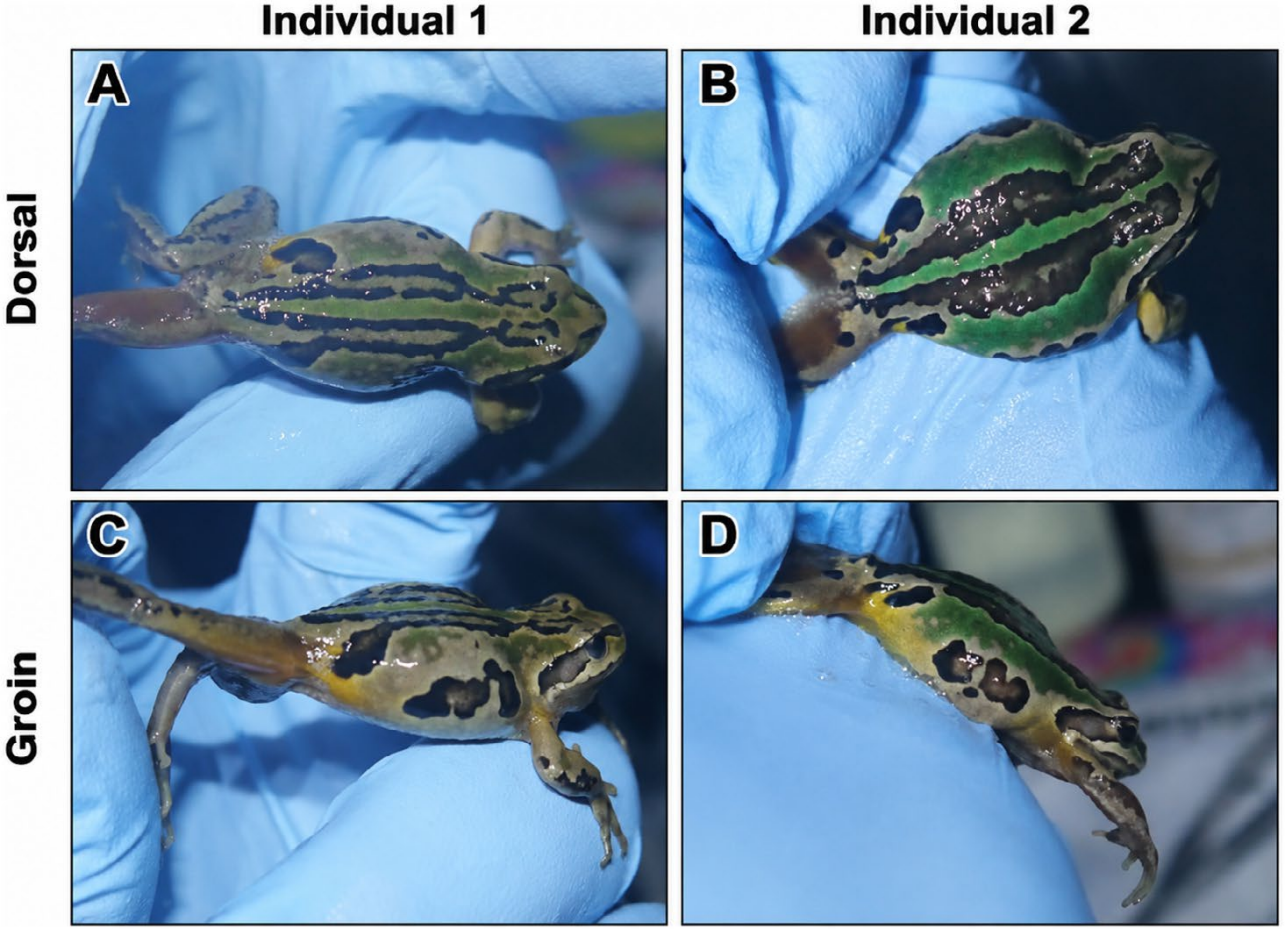
Dorsal and groin views of two representative *Litoria verreauxii alpina* individuals used to illustrate the natural pattern variation employed for photographic identification. (A, C) correspond to the dorsal and groin views, respectively, of Individual 1, and (B, D) correspond to the dorsal and groin views, respectively, of Individual 2. Individual frogs were identified across capture events by comparison of natural markings on the dorsal surface and groin.

We also surveyed egg masses within the breeding pond, with each egg mass defined as a distinct clump of eggs deposited in a single location. Each egg mass was recorded and samples taken only once during the week in which it was first discovered. Egg masses were not counted again during subsequent weekly surveys. Photographs of each egg mass were used to count the number of visible eggs and to estimate the proportion of the mass visible in the photograph and thereby calculate the total number of eggs within the mass. We sampled 4 eggs from different locations on the outside of the egg mass to limit disturbance to the egg mass while increasing the chance of detecting multiple paternity. We stored the eggs in 100% ethanol. Both toe tip clips and sampled eggs were stored at −20°C in the field and −80°C long‐term until DNA extraction was performed.

All work was conducted under approval of the University of Melbourne's Animal Ethics Committee (applications 1814537) and Wildlife Act 1975 Research Authorization permit numbers 10008829.

### 2.3 Parentage Analysis

To assign parentage to egg masses, we genotyped all adult toe clips as well as three eggs per egg mass (165 total eggs); the fourth sampled egg from each mass was retained as a reserve in case additional DNA extraction or genotyping was required. Each egg mass was treated as an individual sampled unit for initial parentage assignment; however, when multiple egg masses were assigned to the same sire and dam, they were interpreted as components of a single clutch, defined here as the total reproductive output of that mating pair. Tissue samples and eggs were sent to Diversity Arrays Technology (DArT: Canberra, Australia) for genotyping using DArTseq. DArTseq uses a combination of DArT complexity reduction methods and next‐generation sequencing platforms (Jaccoud et al. 2001; Sansaloni et al. 2011; Kilian et al. 2012; Cruz et al. 2013). DNA was extracted using the NucleoMag Tissue kit (MACHEREY‐NAGEL). Genomic DNA was digested using the restriction enzymes Pstl and Sphl, and adapters containing sample‐specific barcodes and Illumina sequences were ligated to the resulting fragments. A reduced‐representation subset of fragments was subsequently amplified by PCR and sequenced using an Illumina HiSeq 2500 platform. Sequence reads were processed through a proprietary DArT analytical pipeline to identify high‐confidence single nucleotide polymorphisms (SNPs) and presence/absence variants from variation within homologous fragments, which were then used for downstream genetic analyses.

A dataset of 85,374 SNPs was returned, and these were stringently filtered in R using the RStudio interface (R Core Team 2024; RStudio Team 2024) and the dartR package (Gruber et al. 2018) to 3570 SNP loci that had a call rate of higher than 90% and reproducibility higher than 98%. Following this locus‐level filtering, individuals genotyped at fewer than 40% of retained SNP loci were removed. To minimize redundancy among markers, loci in linkage disequilibrium were filtered using a threshold of $R^{2}>0.2$, such that one SNP from each pair of loci in stronger linkage disequilibrium was removed prior to parentage analyses, leaving 2953 SNPs in the final dataset. From these SNPs and the successfully genotyped individuals, we used the program COLONY 2 (Wang 2004, 2012, 2013a, 2013b; Jones and Wang 2010a, 2010b) to determine parentage of egg masses which implements a full‐pedigree likelihood approach.

Input files were formatted according to COLONY's requirements, with individuals classified as candidate offspring, candidate mothers, or candidate fathers based on developmental stage (egg or adult) and field observations of sexual characteristics. COLONY settings included the assumption that both males and females were polygamous, reflecting the expected reproductive behavior in this species. We also used the full‐likelihood method with long run length and 3 replicate runs to ensure consistency of results. Allele dropout rates at each locus were set at 0.05 with other typing error rates at each locus set at 0.05. The probability of an egg's sire/dam being included among the individuals captured was set at 0.8/0.2 respectively based on observed sex‐biased detection rates in field surveys. The effective population size (*N*_*e*_) was estimated from the reconstructed sibship structure using COLONY's internal methods, which infer *N*_*e*_ based on the number and distribution of full‐ and half‐sibling relationships. COLONY reports *N*_*e*_ under alternative random‐mating and non‐random‐mating model assumptions, and both estimates are presented here as outputs of those internal model settings rather than as separate mating patterns inferred directly from the data. Post‐analysis inferred relationships were filtered based on a posterior probability threshold of 0.95 to retain only high‐confidence assignments. The final pedigree structure was used for downstream analyses, including assessments of reproductive success, relatedness, and family structure within the population.

### 2.4 Statistical Analyses

All statistical tests were performed in R using the RStudio interface (R Core Team 2024; RStudio Team 2024). To investigate reproductive output among mating pairs, we constructed one generalized linear model (GLM) and two generalized linear mixed‐effect models (GLMEs), all with Poisson distributions. These models examined the number of egg masses produced, the number of eggs per mass, and the total number of eggs produced per mating pair. The two GLMEs included individual mating pairs identified in COLONY as a random effect. A binomial distribution was used to model male breeding success. All models were evaluated and no model assumptions were violated.

We used a GLM with a Poisson distribution to test whether the timing within the breeding season influenced the weekly egg‐mass count per mating pair. Following genetic parentage assignment, egg masses were grouped by mating pair and associated with the week in which the egg mass was first discovered. Because each egg mass was recorded only once, the response variable represented the number of spatially distinct egg masses detected for a mating pair during one weekly sampling interval. Because each sampling was one week, this count represents the number of egg masses attributed to a pair per week. This analysis evaluated whether mating pairs were associated with a single egg mass or multiple spatially distinct egg masses during that interval. All egg masses attributed to a given mating pair were detected within the same sampling week, and each mating pair therefore contributed a single observation to the analysis. Week was included as a predictor. This model included all mating pairs for which egg mass data were available, regardless of whether clutch size data were available.

We also tested whether the timing within the breeding season influenced the number of eggs per egg mass. A GLME with a Poisson distribution was used, where the response variable was the egg count per egg mass, the fixed effect was week, and the random effect was mating pair identity to account for mating pairs represented by more than one egg mass. Each egg mass contributed a single egg‐count observation to the analysis. Egg mass identity was therefore not included as a random effect nested within mating pair because there were no repeated observations within individual egg masses from which egg‐mass‐level variance could be estimated. This analysis was restricted to a subset of 34 egg masses for which reliable egg counts could be obtained from photographs.

We conducted a separate GLME with a Poisson distribution to test whether the timing within the breeding season influenced the total number of eggs per unique male–female combination. The response variable was total egg output per mating pair, calculated by summing the egg counts across all egg masses attributed to that pair. The fixed effect was week, and the random effect was mating pair identity. This analysis was limited to 14 mating pairs, omitting mating pairs where total egg counts could not be reliably determined from photographs.

To assess factors influencing male breeding success, we constructed a GLME with a binomial distribution. The response variable was breeding success, defined as a binary outcome where males identified as sires during the breeding season were assigned a value of “1,” and those not identified as sires were assigned a “0.” The fixed effects were breeding season timing (early or late), snout–vent length (SVL), and number of recaptures, and the random effect was individual identity to account for repeated observations.

## 3 Results

### 3.1 Sampling

Over the 6‐week study period, we recorded 477 capture events involving a total of 197 unique individuals (179 males and 18 females). There were 55 egg masses observed ranging in size from approximately 10–550 eggs per egg mass.

### 3.2 Parentage Analysis

DNA extraction and sequencing were successful for 100% (197/197) of all adult frogs and 77.6% (128/165) of eggs with the remaining 41 eggs affected by bacterial contamination. The bacterial contamination was found to be from the genus *Herbaspirillum*, a bacterium known to infect amphibian eggs (Bishop et al. 2021; Davidson et al. 2022). This infection resulted in 10 egg masses having zero eggs genotyped, 2 egg masses with one egg genotyped each, and 43 egg masses with two or more eggs genotyped. The majority of egg masses that showed bacterial contamination were laid earlier in the season (15/55 egg masses at or before week 2 and 2/55 egg masses post week 2). Paternity was assigned to 13.4% of adult males captured (24/179), and maternity was assigned to 16.7% of adult females captured (3/18). Of the 45 egg masses that had at least one egg successfully sequenced, 97.7% (44/45) had a definitive sire identified, while only 13.3% (6/45) egg masses had a definitive dam identified, and 2.2% (1/45) had neither a captured sire nor dam assigned. Low maternity assignment likely reflected the limited number of captured females available as candidate parents, as all sampled adults were successfully genotyped. Among the 24 captured males assigned paternity, 13 sired 1 egg mass, 6 sired 2 egg masses, 3 sired 3 egg masses, 1 sired 4 egg masses, and 1 sired 6 egg masses (Table 1). Among egg masses for which more than one egg was successfully genotyped, all sampled eggs within a mass were assigned to the same sire, providing no evidence of multiple paternity. A complete clutch‐level summary of sire and dam assignments is provided in Table 1.

**TABLE 1:** Reconstructed mating pairs and associated egg masses identified using COLONY.

Egg Mass ID	Sire ID	Dam ID	Number mates per sire	Estimated egg mass size
LvaEM01‐18‐10‐12	Lva73‐18‐10‐12	#1	1	110
LvaEM05‐18‐10‐12	Lva19‐18‐10‐12	#11	1	550
LvaEM06‐18‐10‐12	Lva10‐18‐10‐12^**†**^	#14	3	297
LvaEM07‐18‐10‐12	Lva50‐18‐10‐12	#16	1	—
LvaEM08‐18‐10‐12	Lva32‐18‐10‐12	#17	1	—
LvaEM09‐18‐10‐12	Lva15‐18‐10‐16	#18	1	—
LvaEM10‐18‐10‐12	Lva53‐18‐10‐12	#19	1	—
LvaEM11‐18‐10‐12	Lva77‐18‐10‐12	#2	1	123
LvaEM01‐18‐10‐16	Lva77‐18‐10‐12	#2	1	360
LvaEM02‐18‐10‐16	Lva77‐18‐10‐12	#2	1	210
LvaEM04‐18‐10‐16	Lva77‐18‐10‐12	#2	1	75
LvaEM05‐18‐10‐16	Lva36‐18‐10‐16	#12	1	83
LvaEM06‐18‐10‐16	Lva33‐18‐10‐16	#15	1	95
LvaEM12‐18‐10‐16	*1	#20	1	—
LvaEM13‐18‐10‐16	Lva05‐18‐10‐12	#21	1	—
LvaEM14‐18‐10‐16	Lva07‐18‐10‐22	#22	1	—
LvaEM18‐18‐10‐16	Lva07‐18‐10‐22	#22	1	—
LvaEM15‐18‐10‐16	Lva91‐18‐10‐12	#4^**†**^	1	243
LvaEM17‐18‐10‐16	Lva10‐18‐10‐12^**†**^	#24	3	90
LvaEM01‐18‐10‐19	Lva49‐18‐10‐12	#3	1	139
LvaEM02‐18‐10‐19	Lva49‐18‐10‐12	#3	1	30
LvaEM01‐18‐10‐22	Lva10‐18‐10‐22	#4^**†**^	1	163
LvaEM02‐18‐10‐22	Lva93‐18‐10‐12	#7	1	18
LvaEM01‐18‐11‐05	Lva95‐18‐10‐12	#5	1	140
LvaEM03‐18‐11‐05	Lva26‐18‐10‐12	Lva03‐18‐11‐03	1	120
LvaEM04‐18‐11‐05	Lva26‐18‐10‐12	Lva03‐18‐11‐03	1	—
LvaEM02‐18‐11‐05	Lva87‐18‐10‐12	Lva02‐18‐11‐03	1	115
LvaEM05‐18‐11‐05	Lva87‐18‐10‐12	Lva02‐18‐11‐03	1	133
LvaEM06‐18‐11‐05	Lva87‐18‐10‐12	Lva02‐18‐11‐03	1	170
LvaEM07‐18‐11‐05	Lva10‐18‐10‐12^**†**^	Lva07‐18‐10‐16	3	—
LvaEM01‐18‐11‐09	Lva110‐18‐10‐12	#6	1	55
LvaEM02‐18‐11‐09	Lva110‐18‐10‐12	#6	1	10
LvaEM08‐18‐11‐09	Lva110‐18‐10‐12	#6	1	117
LvaEM09‐18‐11‐09	Lva110‐18‐10‐12	#6	1	78
LvaEM10‐18‐11‐09	Lva110‐18‐10‐12	#6	1	60
LvaEM11‐18‐11‐09	Lva110‐18‐10‐12	#6	1	54
LvaEM03‐18‐11‐09	Lva42‐18‐10‐12	#8	1	38
LvaEM13‐18‐11‐09	Lva42‐18‐10‐12	#8	1	—
LvaEM04‐18‐11‐09	Lva104‐18‐10‐12	#10	1	68
LvaEM07‐18‐11‐09	Lva104‐18‐10‐12	#10	1	58
LvaEM05‐18‐11‐09	Lva04‐18‐11‐09	#13	1	85
LvaEM06‐18‐11‐09	Lva04‐18‐11‐09	#13	1	35
LvaEM12‐18‐11‐09	Lva04‐18‐11‐09	#13	1	47
LvaEM14‐18‐11‐09	Lva36‐18‐10‐12	#23	1	67
LvaEM15‐18‐11‐09	Lva36‐18‐10‐12	#23	1	91

*Note:* Egg mass IDs include the species abbreviation, egg mass number, and sampling date in YY‐MM‐DD format. Sire and dam IDs are shown as either field IDs, which include the species abbreviation, individual ID number and date of first encounter, or inferred parent numbers assigned by COLONY. The number of mates per sire represents the number of individual dams assigned to each male during the 2018 breeding season. Estimated egg mass size was determined from egg counts in photographs. Gray and white shading indicate alternating mating pairs. Individuals marked with † are the only male and female identified as having more than one mate during the 2018 breeding season. The symbols * and # denote inferred sires and dams, respectively, assigned by COLONY.

Estimates of effective population size (*N*_*e*_) were similar under different mating assumptions. Assuming random mating, *N*_*e*_ was estimated at 43 individuals (95% CI: 30–65). Under the assumption of non‐random mating, the estimate was 35 individuals (95% CI: 23–55).

### 3.3 Mating Pairs

Parentage reconstruction identified 28 unique mating pairs across the 45 genotyped egg masses (Table 1). One mating pair involving an inferred, uncaptured male was included in the egg‐mass timing analysis. All egg masses attributed to a given mating pair were detected during the same weekly survey and were estimated to be of the same developmental age. The weekly egg‐mass count per mating pair increased across the breeding season. From week 1 to week 5, the expected number of egg masses per mating pair increased by approximately 138%, where the median egg mass counts increased from 1 (IQR = 1–4) in week 1 to 3 (IQR = 2–6) in week 5 (GLM: Week, *β* = 0.216 ± 0.097 SE, *z* = 2.221, *p* = 0.026; Figure 2A).

**FIGURE 2:**
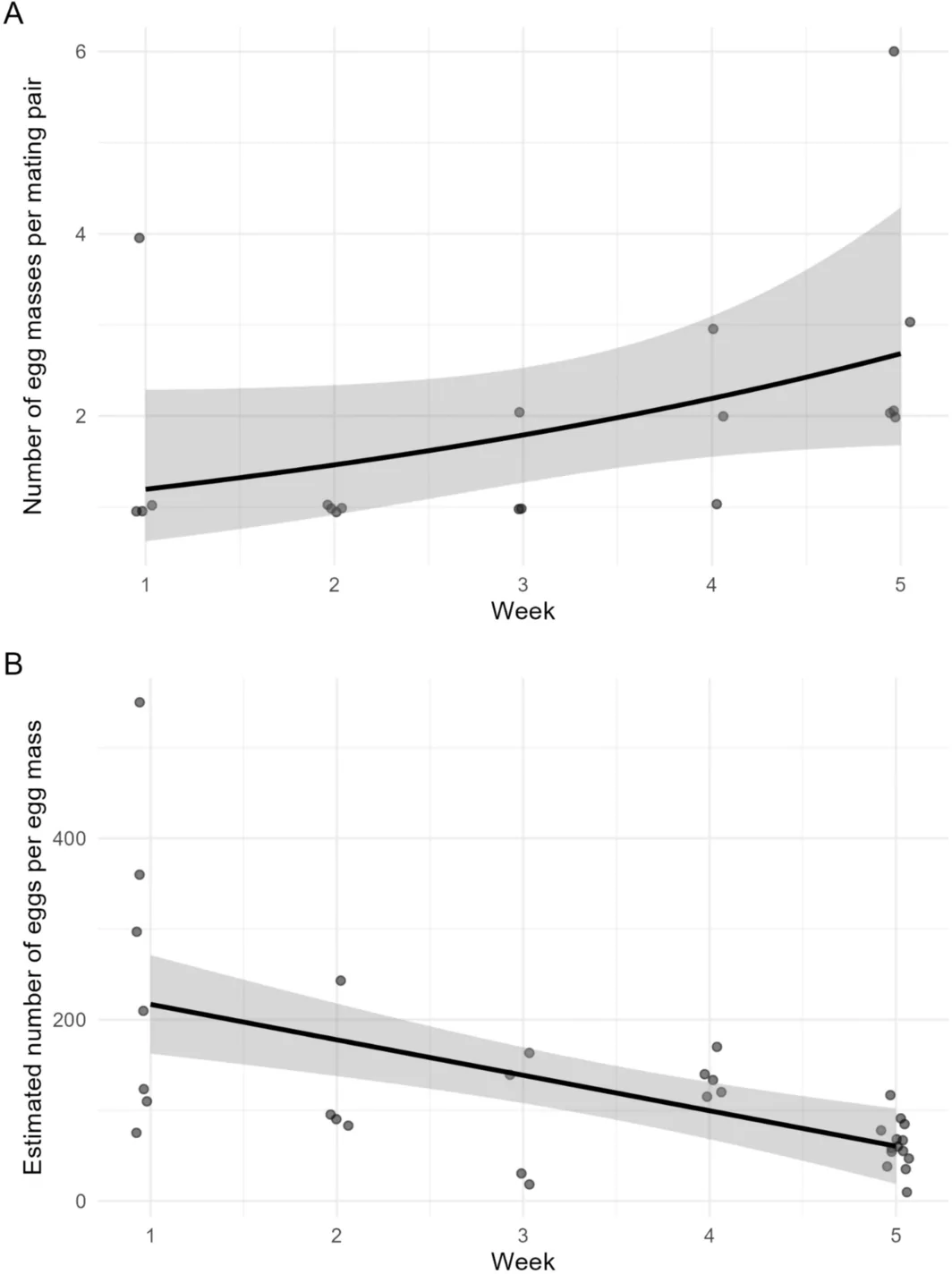
Generalized linear regression plots showing changes in reproductive patterns across the breeding season by week. (A) Number of egg masses produced per mating pair in each weekly spawning event. (B) Estimated number of eggs per egg mass across weeks. In both panels, the solid black line represents predicted values from a Poisson regression model, with shaded areas indicating the 95% confidence intervals.

We next tested whether the timing within the breeding season influenced the number of eggs per egg mass. There was a significant association between week and egg count per mass (GLME: Week, *χ* ^2^_1_ = 10.293, *p* = 0.0013; Random effect: mating pair, variance = 0.3359 ± 0.5796). Mean egg‐mass size declined over time, with egg masses detected in week 1 containing a mean of 246.43 eggs (SD = 169.40), compared with 61.64 eggs (SD = 26.42) in week 5, which is a reduction of approximately 75% (Figure 2B).

We also analyzed whether the timing within the breeding season predicted the total egg output per male–female pair, summed across all egg masses attributed to that pair during the weekly sampling interval. Week did not significantly influence total egg output (GLME: Week, *χ* ^2^_1_ = 0.1823, *p* = 0.6694; Random effect: mating pair, variance = 0.7201 ± 0.8486). On average, pairs produced 239 eggs (range: 18–768), distributed across 2.1 egg masses per pair (range: 1–6).

### 3.4 Male Mating Success

We analyzed whether timing within the breeding season, body size, or number of recaptures affected male reproductive success and found no significant effects. The proportion of successful sires was similar in the early (14.1%) and late (14.0%) breeding season, indicating no seasonal trend in mating success. Across all sampled males, the average snout–vent length (SVL) was 32.7 ± 1.72 mm, and individuals were recaptured an average of 2.5 times (range: 1–6). Variation in body size, capture frequency, or seasonal timing did not significantly predict male reproductive success (GLME: Week, *χ* ^2^_1_ < 0.001, *p* = 0.983; SVL, *χ* ^2^_1_ = 0.009, *p* = 0.967; Recaptures, *χ* ^2^_1_ = 0.003, *p* = 0.949; Random effect: individual, variance = 4436.00 ± 66.61).

During the breeding season, only one captured male was assigned offspring with more than one female, while one inferred, uncaptured female was associated with two different males (Table 1). The male was assigned egg masses with three different females in weeks 1, 2, and 4, and was captured twice, in weeks 1 and 3. The female was inferred to have mated with two different males in weeks 2 and 3.

## 4 Discussion

Throughout the 6‐week breeding season, *L. v. alpina* exhibited a parentage pattern consistent with genetic monogamy, with nearly all assigned individuals linked to offspring from a single reproductive pairing within the sampled data. This was the central and most unexpected result of our study, because the species breeds in communal aggregations where high encounter rates among adults would be expected to increase opportunities for multiple mating. Here, genetic monogamy refers to the reconstructed parentage pattern rather than social monogamy or exclusive behavioral association between males and females. This pattern is striking given the species' breeding ecology. *Litoria v. alpina* forms dense, communal aggregations in shallow ponds over a 6‐to‐8‐week period during the breeding season (Brannelly et al. 2015, 2016, 2024). In such systems, high encounter rates and male‐biased breeding‐site attendance can create opportunities for multiple mating and unequal reproductive success, although realized mating patterns may still depend on female choice, male advertisement, and spatial or temporal structure within the breeding site (Wells 2007; Zamudio et al. 2016; Kelleher et al. 2021). However, despite numerous opportunities for reproductive interaction, as confirmed by capture records indicating that individual males were present for several weeks throughout the season, genetic parentage analysis revealed little evidence of multiple‐partner reproduction. Thus, the realized genetic mating system differed from expectations based on breeding aggregation structure alone, supporting the value of genetic parentage data for revealing mating outcomes that may not be apparent from social or breeding behavior (Avise et al. 2002; O'Brien et al. 2018; de Sá et al. 2020).

These conclusions should be interpreted within the practical constraints of working with an endangered species. Ethical considerations limited sampling up to four eggs per egg mass, but 55 egg masses were genotyped, and a substantial proportion of breeding males were sampled. Parentage could be assigned less completely on the maternal side, which is not unexpected in amphibian breeding studies. At breeding sites, males often predominate because they congregate for longer periods, whereas females may arrive more asynchronously and disperse soon after breeding (Staufer et al. 2023). Although some dams and breeding events may not have been captured, the available data nevertheless support low observed multiple‐partner parentage and limited reproductive skew among the breeders represented here. Low levels of undetected multiple paternity remain possible, particularly given the limited number of eggs genotyped per egg mass and the less than complete assignment of maternity (Myers and Zamudio 2004). Even so, the reconstructed parentage pattern across the sampled egg masses suggests that partner switching was uncommon and that the breeders represented in the dataset contributed relatively little reproductive skew. For this reason, we interpret genetic monogamy here as the dominant reconstructed parentage pattern rather than as definitive evidence that all social pairings were exclusive or that no unsampled multiple paternity occurred.

Only one male (captured and sampled) and one (inferred) female were found to have produced offspring with more than one partner throughout the entire season. These two individuals appear to be exceptions within an otherwise genetically monogamous system. One captured male was assigned offspring with more than one female across weeks 1, 2, and 4, and was also captured in weeks 1 and 3, suggesting prolonged site attendance or repeated returns to the breeding pond. Such extended male presence is common in aggregate‐breeding frogs, where males often remain at breeding sites over multiple nights or weeks (Wheeler and Welsh Jr. 2008; Boyle et al. 2021; Dougherty et al. 2022). However, despite repeated captures of males across the breeding season, recapture frequency did not predict male reproductive success in this population. This suggests that prolonged attendance alone did not generally translate into greater reproductive success or increased reproductive skew. At the same time, some male–female combinations were associated with multiple egg masses, but this most likely reflects the spatial partitioning of reproductive output among multiple egg masses associated with the same spawning event. All egg masses attributed to a given pair were detected during the same weekly survey and estimated to be of the same developmental age. Although the weekly sampling resolution prevented us from determining whether these egg masses were deposited on the same night, the pattern was consistent with pairs distributing their eggs among multiple locations during a single reproductive period. Thus, multiple egg masses attributed to one pair did not necessarily indicate that the individuals mated repeatedly and do not conflict with the overall pattern of genetic monogamy. Similar flexibility is consistent with reproductive plasticity described in other anurans (Byrne and Keogh 2009; Byrne and Roberts 2012; Zamudio et al. 2016), and captive breeding work indicates that *L. v. alpina* can show flexibility in reproductive output under some conditions (Brannelly et al. 2023). The genetic data therefore provide direct evidence of realized parentage among the sampled clutches and are consistent with limited partner switching in the population. The rarity of multiple matings by individual frogs supports the overall pattern of low reproductive skew in this population. In behavioral terms, this pattern suggests that access to mates was not strongly monopolized by a small subset of males, even though some males were repeatedly present at the breeding site throughout the season. Because these conclusions are based on limited offspring sampling and incomplete maternity assignment, they are best interpreted as evidence for low observed reproductive skew among the sampled breeders.

The unexpectedly strong pattern of genetic monogamy might reflect selective mechanisms such as female mate choice, limited mate availability, breeding‐site structure, or a skewed operational sex ratio (Raveh et al. 2014; Mangold et al. 2015; Dittrich and Rödel 2023). Although the mechanisms underlying this pattern remain unresolved, the genetic data show that realized reproductive success was more evenly distributed than expected for a communal‐breeding frog. This contrasts with the expectation that high encounter rates and male‐biased breeding‐site attendance would increase opportunities for multiple mating and variance in male reproductive success. Future work combining parentage analysis with direct behavioral observations could help determine whether this pattern reflects mate choice, breeding‐site structure, temporal variation in reproductive activity, or other aspects of the species' breeding ecology.

Despite the potentially stabilizing mating structure, genetic analysis indicated that the effective population size of this *L. v. alpina* population was 43, below the threshold of 50 individuals commonly associated with an increased risk of short‐term inbreeding depression and reduced adaptive potential (Franklin 1980; Soule 1980; Pérez‐Pereira et al. 2022). Such a small *N*_*e*_ can accelerate the loss of genetic diversity, particularly if compounded by factors like skewed reproductive success or overlapping generations. Since overlapping generations have likely ceased in this system due to *Bd*‐related adult mortality (Scheele et al. 2016), reduced opportunities for reproductive dominance may help buffer against the loss of genetic diversity. In this context, the observed low reproductive skew is behaviourally and genetically relevant because it suggests that realized breeding contribution was distributed across multiple males rather than concentrated among only a few successful individuals, a distinction that can strongly influence effective population size (Hedrick 2005; Hare et al. 2011; Waples 2022).

Several male–female combinations were associated with multiple spatially distinct egg masses detected during the same weekly sampling interval, particularly among pairs detected later in the breeding season. When these egg masses were grouped by male–female combination, the total reproductive output (e.g., total eggs laid) per pair remained consistent across the season. However, later in the season, this output was distributed across a greater number of smaller egg masses. The increase in the number of egg masses per pair, together with the absence of a corresponding change in total egg output, suggests that pairs shifted how they spatially distributed their eggs rather than increasing their overall reproductive investment. This reproductive shift could represent a form of bet‐hedging, whereby individuals distribute their reproductive effort across space or time to reduce the risk of complete failure in variable or unpredictable environments (Chen et al. 2018; Jourdan‐Pineau et al. 2022). Because multiple egg masses attributed to a pair were detected during the same weekly survey and were of a similar estimated developmental age, our results are more consistent with spatial than temporal bet‐hedging. In this system, the most likely selective pressure is seasonal pond drying, which reduces the availability of suitable egg‐laying habitat over time. Although the breeding pond is permanent, it experiences progressive water loss over the season and is used for road maintenance and recreational off‐roading, further disturbing microhabitats and potentially displacing eggs. In alpine regions, snowmelt and rainfall early in the season create ideal spawning conditions, but water levels typically decline into late spring and summer (Carlson et al. 2020). Distributing smaller egg masses across the pond later in the season could buffer against localized desiccation or disturbance events. This pattern suggests that reproductive allocation may shift within a breeding season even when total output per pair remains relatively stable.

Additional selective pressures could also contribute to this pattern. Several early‐season egg masses in this study showed infection with DNA sequence matching *Herbaspirillum*, a bacterium known to infect amphibian eggs (Bishop et al. 2021; Davidson et al. 2022; Brannelly et al. 2025). Although infection was detected earlier in the season, pathogen risk might promote spatial bet‐hedging within a breeding event to reduce the risk of total reproductive failure. Similar strategies have been observed in other amphibians: for example, *Isthmohyla calypsa* (formerly *Hyla calypsa* ) varies its egg‐mass size in response to seasonal increases in fly predation, laying more, smaller egg‐masses later in the season to avoid complete reproductive loss (Lips 2001). Thus, the observed shift in *L. v. alpina* could reflect a flexible reproductive response to multiple interacting stressors including habitat degradation, pond drying, and pathogen risk, that shape breeding success in unpredictable alpine environments.

In this study, male *L. v. alpina* gained no reproductive advantage from increased body size, which is surprising given that male size is often a strong predictor of mating success in many frog species (Tsuji 2004; Luo et al. 2014; Dittrich et al. 2018). While male body size often influences male reproductive success, there are some amphibian species where size is less important, like *Dryophytes versicolor* (formerly *Hyla versicolor* ), which shows random mating with regard to size (Höbel et al. 2022). Similarly, we found no significant association between male recapture frequency and mating success. In other species, such as *Dryophytes cinerea* (formerly *Hyla cinerea* ), site attendance can weakly predict mating success (Gerhardt et al. 1987), but *L. v. alpina* appears to rely on alternative mate selection mechanisms. Our results suggest that female mate choice in *L. v. alpina* is not driven by size or site attendance and might instead rely on other behavioral or physiological cues. Laboratory and field studies show that infection with the fungal pathogen *Bd* can alter male *L. v. alpina* coloration (Brannelly et al. 2025), potentially affecting female mate choice. Together, these results indicate that commonly assumed predictors of male reproductive success, such as size and site attendance, do not explain mating outcomes in this population.

More broadly, our study shows that genetic parentage analysis can reveal unexpected mating‐system structure in amphibians (Avise et al. 2002; O'Brien et al. 2018; de Sá et al. 2020), including species whose social breeding ecology may create opportunities for multiple mating and reproductive skew (Wells 2007; Zamudio et al. 2016). By linking mating structure, male reproductive success, seasonal reproductive allocation, and effective population size, these results highlight the value of integrating behavioral ecology and genetic data when evaluating reproduction in disease‐impacted wildlife populations. Although conservation applications are not the primary focus of this study, the results also suggest that maintaining multiple viable egg‐laying sites across the breeding season could help preserve reproductive opportunities and support variation in reproductive contribution in disease‐impacted alpine frog populations.

## Author Contributions

**Alexander S. Wendt:** data curation (equal), formal analysis (lead), funding acquisition (supporting), investigation (lead), methodology (lead), validation (lead), visualization (lead), writing – original draft (lead). **Anthony W. Waddle:** data curation (equal), investigation (equal), writing – review and editing (equal). **Marcus A. Hough:** data curation (equal), investigation (equal), writing – review and editing (equal). **Lee Berger:** resources (equal), supervision (supporting), writing – review and editing (equal). **Lee F. Skerratt:** resources (supporting), supervision (supporting), writing – review and editing (equal). **Laura A. Brannelly:** conceptualization (lead), data curation (lead), formal analysis (supporting), funding acquisition (lead), investigation (lead), methodology (lead), project administration (lead), resources (lead), supervision (lead), writing – original draft (equal), writing – review and editing (equal).

## Funding

The study was funded by the American Australian Association, the Holsworth Wildlife Research Endowment—Equity Trustees Charitable Foundation, the Ecological Society of Australia, the Australian Research Council (DE180101395), the Australian Academy of Science (Max Day Environmental Science Fellowship), Bioplatforms Australia as part of the Threatened Species Initiative, and the Dr. Albert Shimmins Fund.

## Ethics Statement

All work was conducted under approval of the University of Melbourne's Animal Ethics Committee (applications 1814537) and Wildlife Act 1975 Research Authorization permit numbers 10008829.

## Conflicts of Interest

The authors declare no conflicts of interest.

## Data Availability

The data and analysis files supporting the findings of this study are openly available on Figshare at https://doi.org/10.26188/32109115. These files include the R code, raw data, and associated input files used to analyze reproductive output, parentage, and mating patterns.

## References

[ece374510-bib-0001] Addis, B. R. , W. H. Lowe , B. R. Hossack , and F. W. Allendorf . 2015. “Population Genetic Structure and Disease in Montane Boreal Toads: More Heterozygous Individuals Are More Likely to be Infected With Amphibian Chytrid.” Conservation Genetics 16: 833–844. 10.1007/s10592-015-0704-6.

[ece374510-bib-0002] Angom, S. , C. Tuboi , M. G. Ullah Ghazi , M. G. U. Ghazi , R. Badola , and S. A. Hussain . 2020. “Demographic and Genetic Structure of a Severely Fragmented Population of the Endangered HOG Deer ( *Axis porcinus* ) in the Indo‐Burma Biodiversity Hotspot.” PLoS One 15: e0210382. 10.1371/journal.pone.0210382. PMC7004368 32027650

[ece374510-bib-0003] Anstis, M. 2017. Tadpoles and Frogs of Australia. 2nd ed. New Holland Publishers.

[ece374510-bib-0005] Avise, J. C. , A. G. Jones , D. E. Walker , J. A. DeWoody , and collaborators . 2002. “Genetic Mating Systems and Reproductive Natural Histories of Fishes: Lessons for Ecology and Evolution.” Annual Review of Genetics 36: 19–45. 10.1146/annurev.genet.36.030602.090831. 12429685

[ece374510-bib-0006] Baker, P. J. , S. M. Funk , M. W. Bruford , and S. Harris . 2004. “Polygynandry in a Red Fox Population: Implications for the Evolution of Group Living in Canids?” Behavioral Ecology 15: 766–778. 10.1093/beheco/arh077.

[ece374510-bib-0007] Beranek, C. T. , C. Maynard , C. McHenry , J. Clulow , and M. Mahony . 2022. “Identifying a Limiting Factor in the Population Dynamics of a Threatened Amphibian: The Influence of Extended Female Maturation on Operational Sex Ratio.” Austral Ecology 47: 239–250. 10.1111/aec.13098.

[ece374510-bib-0008] Bishop, C. , E. Jurga , and L. Graham . 2021. “Patterns of Bacterial Diversity in Embryonic Capsules of the Spotted Salamander *Ambystoma maculatum* : An Expanding View of a Symbiosis.” FEMS Microbiology Ecology 97: fiab128. 10.1093/femsec/fiab128. 34482407

[ece374510-bib-0009] Boyle, C. M. , E. H. Z. Gourevitch , and J. R. Downie . 2021. “Breeding Site Attendance and Breeding Success in *Phyllomedusa trinitatis* (Anura: Phyllomedusidae).” Phyllomedusa: Journal of Herpetology 20: 53–66. 10.11606/ISSN.2316-9079.V20I1P53-66.

[ece374510-bib-0010] Brannelly, L. , B. Scheele , and L. Grogan . 2020. “Disease and the Endangered Alpine Tree Frog: Bridging Research, Conservation and Management.” In Strategies for Conservation Success in Herpetology, edited by S. Walls and K. O'Donnell . Society for the Study of Amphibians and Reptiles Herpetological Conservation Series.

[ece374510-bib-0011] Brannelly, L. A. , L. Berger , and L. F. Skerratt . 2014. “Comparison of Three Widely Used Marking Techniques for Adult Anuran Species * Litoria verreauxii alpina* .” Herpetological Conservation and Biology 9: 428–435.

[ece374510-bib-0013] Brannelly, L. A. , D. A. Hunter , D. Lenger , B. C. Scheele , L. F. Skerratt , and L. Berger . 2015. “Dynamics of Chytridiomycosis During the Breeding Season in an Australian Alpine Amphibian.” PLoS One 10: e0143629. 10.1371/journal.pone.0143629. PMC4668081 26629993

[ece374510-bib-0014] Brannelly, L. A. , D. A. Hunter , L. F. Skerratt , et al. 2016. “Chytrid Infection and Post‐Release Fitness in the Reintroduction of an Endangered Alpine Tree Frog.” Animal Conservation 19: 153–162. 10.1111/acv.12230.

[ece374510-bib-0015] Brannelly, L. A. , B. C. Scheele , and L. F. Grogan . 2024. “Disease and the Endangered Alpine Tree Frog: Bridging Research, Conservation and Management.” In Strategies for Conservation Success in Herpetology, edited by S. C. Walls and K. M. O'Donnell . Society for the Study of Amphibians and Reptiles, Herpetological Conservation Series.

[ece374510-bib-0016] Brannelly, L. A. , P. Sharma , and D. K. Wallace . 2023. “Captive Breeding in the Endangered Alpine Tree Frog, * Litoria verreauxii alpina* .” PeerJ 11: e15179. 10.7717/peerj.15179. PMC10124539 37101793

[ece374510-bib-0017] Brannelly, L. A. , D. K. Wallace , A. S. Wendt , et al. 2025. “Devastating Disease Can Cause Increased Breeding Effort and Success That Improves Population Resilience.” Open Biology 15: 240385. 10.1098/rsob.240385. PMC12115809 40425049

[ece374510-bib-0020] Briggs, C. J. , V. T. Vredenburg , R. A. Knapp , and L. J. Rachowicz . 2005. “Investigating the Population‐Level Effects of Chytridiomycosis: An Emerging Infectious Disease of Amphibians.” Ecology 86: 3149–3159. 10.1890/04-1428.

[ece374510-bib-0021] Brown, J. L. , V. Morales , and K. Summers . 2010. “A Key Ecological Trait Drove the Evolution of Biparental Care and Monogamy in an Amphibian.” American Naturalist 175: 436–446. 10.1086/650727. 20180700

[ece374510-bib-0022] Byrne, P. G. , and J. S. Keogh . 2009. “Extreme Sequential Polyandry Insures Against Nest Failure in a Frog.” Proceedings of the Royal Society B: Biological Sciences 276: 115–120. 10.1098/rspb.2008.0794. PMC2614246 18782745

[ece374510-bib-0023] Byrne, P. G. , and J. D. Roberts . 2012. “Evolutionary Causes and Consequences of Sequential Polyandry in Anuran Amphibians.” Biological Reviews 87: 209–228. 10.1111/j.1469-185X.2011.00191.x. 21740503

[ece374510-bib-0024] Carlson, B. Z. , M. Hébert , C. van Reeth , et al. 2020. “Monitoring the Seasonal Hydrology of Alpine Wetlands in Response to Snow Cover Dynamics and Summer Climate: A Novel Approach With Sentinel‐2.” Remote Sensing 12: 1959. 10.3390/rs12121959.

[ece374510-bib-0026] Chen, W. , L. Peng , L. Jiang , et al. 2018. “High Altitude Frogs (*Rana kukonoris*) Adopt a Diversified Bet‐Hedging Strategy in the Face of Environmental Unpredictability.” Asian Herpetological Research 9: 43–49. 10.16373/j.cnki.ahr.170073.

[ece374510-bib-0027] Cohas, A. , and D. Allainé . 2009. “Social Structure Influences Extra‐Pair Paternity in Socially Monogamous Mammals.” Biology Letters 5: 313–316. 10.1098/rsbl.2008.0760. PMC2679913 19324647

[ece374510-bib-0028] Cooper, J. D. , P. M. Waser , E. C. Hellgren , T. M. Gabor , and J. A. DeWoody . 2011. “Is Sexual Monomorphism a Predictor of Polygynandry? Evidence From a Social Mammal, the Collared Peccary.” Behavioral Ecology and Sociobiology 65: 775–785. 10.1007/s00265-010-1081-2.

[ece374510-bib-0029] Crawford‐Ash, J. , and J. J. L. Rowley . 2021. “Photo Identification of Individual Blue Mountains Tree Frogs ( *Litoria citropa* ).” Herpetological Notes 14: 803–808.

[ece374510-bib-0030] Cruz, V. M. V. , A. Kilian , and D. A. Dierig . 2013. “Development of DArT Marker Platforms and Genetic Diversity Assessment of the U.S. Collection of the New Oilseed Crop Lesquerella and Related Species.” PLoS One 8: e64062. 10.1371/journal.pone.0064062. PMC3665832 23724020

[ece374510-bib-0031] Davidson, M. J. , R. Bushell , R. Ploeg , et al. 2022. “Embryo Mortality in a Captive‐Bred, Critically Endangered Amphibian.” Diseases of Aquatic Organisms 152: 73–83. 10.3354/DAO03706. 36453456

[ece374510-bib-0032] de Sá, F. P. , R. C. Consolmagno , P. Muralidhar , C. A. Brasileiro , K. R. Zamudio , and C. F. B. Haddad . 2020. “Unexpected Reproductive Fidelity in a Polygynous Frog.” Science Advances 6: eaay1539. 10.1126/sciadv.aay1539. PMC7423391 32851153

[ece374510-bib-0033] Dittrich, C. , and M. O. Rödel . 2023. “Drop Dead! Female Mate Avoidance in an Explosively Breeding Frog.” Royal Society Open Science 10: 230742. 10.1098/rsos.230742. PMC10565404 37830023

[ece374510-bib-0034] Dittrich, C. , A. Rodríguez , O. Segev , et al. 2018. “Temporal Migration Patterns and Mating Tactics Influence Size‐Assortative Mating in *Rana temporaria* .” Behavioral Ecology 29: 418–428. 10.1093/beheco/arx188. PMC5873255 29622935

[ece374510-bib-0035] Dougherty, R. P. , R. M. Pulica , and M. S. Caldwell . 2022. “Multi‐Night Territorial Behavior, Chorus Attendance, and Mating Success in Red‐Eyed Treefrogs.” Ethology 128: 608–619. 10.1111/eth.13321.

[ece374510-bib-0036] Elsner‐Gearing, F. , P. Kretzschmar , S. Shultz , et al. 2024. “Admixture and Reproductive Skew Shape the Conservation Value of Ex Situ Populations of the Critically Endangered Eastern Black Rhino.” Conservation Genetics 25: 897–910. 10.1007/s10592-024-01611-z.

[ece374510-bib-0037] Fitzpatrick, C. L. , and M. R. Servedio . 2017. “Male Mate Choice, Male Quality, and the Potential for Sexual Selection on Female Traits Under Polygyny.” Evolution 71: 174–183. 10.1111/evo.13107. 27804119

[ece374510-bib-0038] Franklin, I. R. 1980. “Evolutionary Changes in Small Populations.” In Conservation Biology: An Evolutionary‐Ecological Prospective, edited by M. E. Soule and B. A. Wilcox , 135–149. Sinauer Associates.

[ece374510-bib-0039] Gerhardt, H. C. , R. E. Daniel , S. A. Perrill , and S. Schramm . 1987. “Mating Behaviour and Male Mating Success in the Green Treefrog.” Animal Behaviour 35: 1490–1503. 10.1016/S0003-3472(87)80021-0.

[ece374510-bib-0040] Gillespie, G. , W. Osborne , and N. McElhinney . 1995. The Conservation Status of Frogs in the Australian Alps: A Review.

[ece374510-bib-0041] Godwin, J. L. , A. J. Lumley , Ł. Michalczyk , O. Y. Martin , and M. J. G. Gage . 2020. “Mating Patterns Influence Vulnerability to the Extinction Vortex.” Global Change Biology 26: 4226–4239. 10.1111/gcb.15186. 32558066

[ece374510-bib-0042] Gould, J. , and J. W. Valdez . 2023. “Dating on Your Level: Assortative Mating Based on Body Condition in an Amphibian.” Ethology Ecology and Evolution 35: 1–15. 10.1080/03949370.2021.1988718.

[ece374510-bib-0044] Gruber, B. , P. J. Unmack , O. F. Berry , and A. Georges . 2018. “Dartr: An r Package to Facilitate Analysis of SNP Data Generated From Reduced Representation Genome Sequencing.” Molecular Ecology Resources 18: 691–699. 10.1111/1755-0998.12745. 29266847

[ece374510-bib-0045] Hare, M. P. , L. Nunney , M. K. Schwartz , et al. 2011. “Understanding and Estimating Effective Population Size for Practical Application in Marine Species Management.” Conservation Biology 25: 438–449. 10.1111/j.1523-1739.2010.01637.x. 21284731

[ece374510-bib-0046] Hedrick, P. 2005. “Large Variance in Reproductive Success and the ne/N Ratio.” Evolution 59: 1596–1599. 10.1111/j.0014-3820.2005.tb01809.x. 16153045

[ece374510-bib-0047] Höbel, G. , O. Feagles , A. Moore , M. Reichert , and K. Stratman . 2022. “Random Mating for Body Size Despite Fitness Benefits of Size‐Assortative Mating in a Treefrog.” Ethology 128: 580–590. 10.1111/eth.13316.

[ece374510-bib-0048] Holman, L. , and H. Kokko . 2013. “The Consequences of Polyandry for Population Viability, Extinction Risk and Conservation.” Philosophical Transactions of the Royal Society, B: Biological Sciences 368: 20120053. 10.1098/rstb.2012.0053. PMC3576587 23339244

[ece374510-bib-0049] Hoogland, J. L. , R. Trott , and S. R. Keller . 2019. “Polyandry and Polygyny in a Social Rodent: An Integrative Perspective Based on Social Organization, Copulations, and Genetics.” Frontiers in Ecology and Evolution 7: 3. 10.3389/fevo.2019.00003.

[ece374510-bib-0050] Hunter, D. , W. Osborne , and M. Smith . 1998. Distribution and Abundance of the Alpine Tree Frog (Litoria verreauxii alpina) in the Australian Alps National Parks. Report to NSW NPWS, Applied Ecology Resource Group, University of Canberra.

[ece374510-bib-0051] Jaccoud, D. , K. Peng , D. Feinstein , and A. Kilian . 2001. “Diversity Arrays: A Solid State Technology for Sequence Information Independent Genotyping.” Nucleic Acids Research 29: e25. 10.1093/nar/29.4.e25. PMC29632 11160945

[ece374510-bib-0052] Jones, O. R. , and J. Wang . 2010a. “Molecular Marker‐Based Pedigrees for Animal Conservation Biologists.” Animal Conservation 13: 26–34. 10.1111/j.1469-1795.2009.00324.x.

[ece374510-bib-0053] Jones, O. R. , and J. Wang . 2010b. “COLONY: A Program for Parentage and Sibship Inference From Multilocus Genotype Data.” Molecular Ecology Resources 10: 551–555. 10.1111/j.1755-0998.2009.02787.x. 21565056

[ece374510-bib-0054] Jourdan‐Pineau, H. , P. A. Crochet , and P. David . 2022. “Bimodal Breeding Phenology in the Parsley Frog *Pelodytes punctatus* as a Bet‐Hedging Strategy in an Unpredictable Environment Despite Strong Priority Effects.” Peer Community Journal 2: e57. 10.24072/pcjournal.176.

[ece374510-bib-0055] Kelleher, S. R. , B. C. Scheele , A. J. Silla , et al. 2021. “Disease Influences Male Advertisement and Mating Outcomes in a Critically Endangered Amphibian.” Animal Behaviour 173: 145–157. 10.1016/j.anbehav.2021.01.007.

[ece374510-bib-0056] Kenyon, N. , A. D. Phillott , and R. A. Alford . 2009. “Evaluation of the Photographic Identification Method (Pim) as a Tool to Identify Adult *Litoria genimaculata* (Anura: Hylidae).” Herpetological Conservation and Biology 4: 403–410.

[ece374510-bib-0057] Kilian, A. , P. Wenzl , E. Huttner , et al. 2012. “Diversity Arrays Technology: A Generic Genome Profiling Technology on Open Platforms.” Methods in Molecular Biology 888: 67–89. 10.1007/978-1-61779-870-2_5. 22665276

[ece374510-bib-0058] Kuehnel, S. , and A. Kupfer . 2012. “Sperm Storage in Caecilian Amphibians.” Frontiers in Zoology 9: 12. 10.1186/1742-9994-9-12. PMC3418217 22672478

[ece374510-bib-0059] Kühnel, S. , S. Reinhard , and A. Kupfer . 2010. “Evolutionary Reproductive Morphology of Amphibians: An Overview.” Bonn Zoological Bulletin 57: 119–126.

[ece374510-bib-0060] Lampo, M. , C. Señaris , K. González , and O. Ballestas . 2023. “Smaller Size of Harlequin Toads From Populations Long Exposed to the Fungal Disease Chytridiomycosis.” Biotropica 55: 699–705. 10.1111/btp.13220.

[ece374510-bib-0061] Lips, K. R. 2001. “Reproductive Trade‐Offs and Bet‐Hedging in *Hyla calypsa* , a Neotropical Treefrog.” Oecologia 128: 509–518. 10.1007/s004420100687. 28547396

[ece374510-bib-0062] Luo, Z. , C. Li , H. Wang , et al. 2014. “Mutual Mate Choice in the Asiatic Toad, *Bufo gargarizans* , Exerts Stabilizing Selection on Body Size.” Chinese Science Bulletin 59: 38–45. 10.1007/s11434-013-0005-8.

[ece374510-bib-0063] Mangold, A. , K. Trenkwalder , M. Ringler , W. Hödl , and E. Ringler . 2015. “Low Reproductive Skew Despite High Male‐Biased Operational Sex Ratio in a Glass Frog With Paternal Care.” BMC Evolutionary Biology 15: 181. 10.1186/s12862-015-0469-z. PMC4558732 26334630

[ece374510-bib-0064] Myers, E. M. , and K. R. Zamudio . 2004. “Multiple Paternity in an Aggregate Breeding Amphibian: The Effect of Reproductive Skew on Estimates of Male Reproductive Success.” Molecular Ecology 13: 1951–1963. 10.1111/j.1365-294X.2004.02208.x. 15189216

[ece374510-bib-0065] O'Brien, D. M. , J. S. Keogh , A. J. Silla , and P. G. Byrne . 2018. “The Unexpected Genetic Mating System of the Red‐Backed Toadlet ( *Pseudophryne coriacea* ): A Species With Prolonged Terrestrial Breeding and Cryptic Reproductive Behaviour.” Molecular Ecology 27: 3001–3015. 10.1111/mec.14737. 29862607

[ece374510-bib-0066] Office of Environment and Heritage (NSW) . 2012. New South Wales State of the Environment 2012.

[ece374510-bib-0067] Osborne, W. , D. Hunter , and G. Hollis . 1999. “Population Declines and Range Contraction in Australian Alpine Frogs.” In Declines and Disappearances of Australian Frogs, edited by A. Campbell , 145–157. Environment Australia.

[ece374510-bib-0068] Pérez‐Pereira, N. , J. Wang , H. Quesada , and A. Caballero . 2022. “Prediction of the Minimum Effective Size of a Population Viable in the Long Term.” Biodiversity and Conservation 31: 2763–2780. 10.1007/s10531-022-02456-z.

[ece374510-bib-0069] Ptak, S. E. , and M. Lachmann . 2003. “On the Evolution of Polygyny: A Theoretical Examination of the Polygyny Threshold Model.” Behavioral Ecology 14: 201–211. 10.1093/beheco/14.2.201.

[ece374510-bib-0070] R Core Team . 2024. R: A Language and Environment for Statistical Computing.

[ece374510-bib-0071] Raveh, S. , S. Sutalo , K. E. Thonhauser , et al. 2014. “Female Partner Preferences Enhance Offspring Ability to Survive an Infection.” BMC Evolutionary Biology 14: 14. 10.1186/1471-2148-14-14. PMC3905909 24450606

[ece374510-bib-0072] Reding, L. 2015. “Increased Hatching Success as a Direct Benefit of Polyandry in Birds.” Evolution; International Journal of Organic Evolution 69: 264–270. 10.1111/evo.12553. 25333786

[ece374510-bib-0073] Reuther, K. D. 2009. Early Invasion Dynamics of the Japanese Pavement Ant, *Tetramorium tsushimae* , in the St. Louis, Missouri Metropolitan Area. Saint Louis University.

[ece374510-bib-0074] RStudio Team . 2024. RStudio: Integrated Development Environment for R.

[ece374510-bib-0075] Rueda‐Solano, L. A. , F. Vargas‐Salinas , J. L. Pérez‐González , et al. 2022. “Mate‐Guarding Behaviour in Anurans: Intrasexual Selection and the Evolution of Prolonged Amplexus in the Harlequin Toad *Atelopus laetissimus* .” Animal Behaviour 185: 127–142. 10.1016/j.anbehav.2021.12.003.

[ece374510-bib-0076] Sansaloni, C. , C. Petroli , D. Jaccoud , et al. 2011. “Diversity Arrays Technology (DArT) and Next‐Generation Sequencing Combined: Genome‐Wide, High Throughput, Highly Informative Genotyping for Molecular Breeding of Eucalyptus.” BMC Proceedings 5, no. Suppl 7: P54. 10.1186/1753-6561-5-s7-p54.

[ece374510-bib-0077] Schaedelin, F. C. , W. F. D. van Dongen , and R. H. Wagner . 2015. “Mate Choice and Genetic Monogamy in a Biparental, Colonial Fish.” Behavioral Ecology 26: 782–788. 10.1093/beheco/arv011. PMC4433329 26023276

[ece374510-bib-0078] Scheele, B. C. , D. A. Hunter , S. C. Banks , et al. 2016. “High Adult Mortality in Disease‐Challenged Frog Populations Increases Vulnerability to Drought.” Journal of Animal Ecology 85: 1453–1460. 10.1111/1365-2656.12569. 27380945

[ece374510-bib-0079] Scheele, B. C. , D. A. Hunter , L. F. Skerratt , L. A. Brannelly , and D. A. Driscoll . 2015. “Low Impact of Chytridiomycosis on Frog Recruitment Enables Persistence in Refuges Despite High Adult Mortality.” Biological Conservation 182: 36–43. 10.1016/j.biocon.2014.11.032.

[ece374510-bib-0080] Sever, D. M. 2002. “Female Sperm Storage in Amphibians.” Journal of Experimental Zoology 292: 165–179. 10.1002/jez.1152. 11754032

[ece374510-bib-0081] Shuster, S. M. 2009. “Sexual Selection and Mating Systems.” Proceedings of the National Academy of Sciences 106: 10009–10016. 10.1073/pnas.0901132106. PMC2702802 19528645

[ece374510-bib-0082] Singh, V. K. , B. D. Joshi , A. Singh , et al. 2022. “Genetic Diversity and Population Structure of the Northern Red Muntjac (*Muntiacus vaginalis*) in Indian Himalayan Region.” Mammalian Biology 102: 537–544. 10.1007/s42991-022-00254-2.

[ece374510-bib-0084] Soule, M. E. 1980. “Thresholds for Survival: Maintaining Fitness and Evolutionary Potential.” In Conservation Biology: An Evolutionary‐Ecological Perspective, edited by M. E. Soulé and B. M. Wilcox , 151–170. Sinauer.

[ece374510-bib-0085] Staufer, M. , S. Burgstaller , A. Horvath , and L. Landler . 2023. “Temporal and Spatial Variations in Local Sex Ratios in a Suburban Population of the European Green Toad *Bufotes viridis* .” BMC Ecology and Evolution 23: 6. 10.1186/s12862-023-02106-0. PMC10024452 36932330

[ece374510-bib-0086] Tennessen, J. A. , and K. R. Zamudio . 2003. “Early Male Reproductive Advantage, Multiple Paternity and Sperm Storage in an Amphibian Aggregate Breeder.” Molecular Ecology 12: 1567–1576. 10.1046/j.1365-294X.2003.01830.x. 12755884

[ece374510-bib-0087] Tsuji, H. 2004. “Reproductive Ecology and Mating Success of Male *Limnonectes kuhlii* , a Fanged Frog From Taiwan.” Herpetologica 60: 155–167. 10.1655/02-10.

[ece374510-bib-0088] Vági, B. , and T. Székely . 2023. “Diversity of Reproductive Strategies in the Amphibia.” In Evolutionary Ecology of Amphibians, edited by G. Moreno‐Rueda and M. Comas , 1st ed., 157–174. CRC Press.

[ece374510-bib-0089] Vági, B. , Z. Végvári , A. Liker , R. P. Freckleton , and T. Székely . 2020. “Climate and Mating Systems as Drivers of Global Diversity of Parental Care in Frogs.” Global Ecology and Biogeography 29: 1373–1386. 10.1111/geb.13113.

[ece374510-bib-0090] Vieites, D. R. , S. Nieto‐Romá , M. Barluenga , et al. 2004. “Post‐Mating Clutch Piracy in an Amphibian.” Nature 431: 305–308. 10.1038/nature02879. 15372032

[ece374510-bib-0091] Wang, J. 2004. “Sibship Reconstruction From Genetic Data With Typing Errors.” Genetics 166: 1963–1979. 10.1534/genetics.166.4.1963. PMC1470831 15126412

[ece374510-bib-0092] Wang, J. 2012. “Computationally Efficient Sibship and Parentage Assignment From Multilocus Marker Data.” Genetics 191: 183–194. 10.1534/genetics.111.138149. PMC3338259 22367033

[ece374510-bib-0093] Wang, J. 2013a. “An Improvement on the Maximum Likelihood Reconstruction of Pedigrees From Marker Data.” Heredity (Edinb) 111: 165–174. 10.1038/hdy.2013.34. PMC3716262 23612692

[ece374510-bib-0094] Wang, J. 2013b. “A Simulation Module in the Computer Program Colony for Sibship and Parentage Analysis.” Molecular Ecology Resources 13: 734–739. 10.1111/1755-0998.12106. 23615269

[ece374510-bib-0095] Wang, X. , S. Liu , Y. Yang , et al. 2020. “Genetic Evidence for the Mating System and Reproductive Success of Black Sea Bream ( *Acanthopagrus schlegelii* ).” Ecology and Evolution 10: 4483–4494. 10.1002/ece3.6215. PMC7246203 32489612

[ece374510-bib-0096] Waples, R. S. 2022. “What Is *ne*, Anyway?” Journal of Heredity 113: 371–379. 10.1093/jhered/esac023. 35532202

[ece374510-bib-0097] Wells, K. D. 2007. The Ecology and Behavior of Amphibians. University of Chicago Press.

[ece374510-bib-0098] Wheeler, C. A. , and H. H. Welsh Jr. 2008. “Mating Strategy and Breeding Patterns of the Foothill Yellow‐Legged Frog ( *Rana boylii* ).” Herpetological Conservation and Biology 3: 128–142.

[ece374510-bib-0099] Winternitz, J. C. , M. Promerova , R. Polakova , et al. 2015. “Effects of Heterozygosity and MHC Diversity on Patterns of Extra‐Pair Paternity in the Socially Monogamous Scarlet Rosefinch.” Behavioral Ecology and Sociobiology 69: 459–469. 10.1007/s00265-014-1858-9.

[ece374510-bib-0100] Zamudio, K. R. , R. C. Bell , R. C. Nali , C. F. B. Haddad , and C. P. A. Prado . 2016. “Polyandry, Predation, and the Evolution of Frog Reproductive Modes.” American Naturalist 188: S41–S61. 10.1086/687547. 27513910

